# Attitudes Toward Mental Illness Among Medical Students: A Cross-Sectional Analysis of Sociodemographic and Experiential Factors

**DOI:** 10.1192/j.eurpsy.2026.11972

**Published:** 2026-07-31

**Authors:** G. Chakchouk, N. Regaieg, F. Guermazi, Y. Trabelsi, I. Feki, J. Masmoudi, I. Baati

**Affiliations:** Department of Psychiatry A, Hedi Chaker Hospital, Sfax, Tunisia

## Abstract

**Introduction:**

Stigmatizing attitudes towards mental illness and psychiatry within medical students can influence their future clinical behavior and the quality of care provided to patients.

**Objectives:**

This study aimed to evaluate medical students’ attitudes toward mental illness and to determine the association between these attitudes and their sociodemographic characteristics, personal or familial mental health experience, and exposure to psychiatric training.

**Methods:**

A cross-sectional study was conducted among medical students enrolled at the medical faculty of Sfax, Tunisia from February to October 2025. Participants completed via a Google form survey a questionnaire including sociodemographic characteristics and personal experiences with mental health.Stigmatizing attitudes were assessed using the validated French version of the Mental Illness Clinicians’ Attitudes (MICA V2) scale destined for medical students.

**Results:**

The number of participants was 81. Most students were in their fourth year (34.6%). Regarding mental health experience: 28.4% had previously consulted a mental health professional, 12.3% had taken psychotropic medication. Within our sample, 27.8% students had a close family member with a psychiatric disorder and 46.3% reported having friends with psychiatric history. 76.9% of the students had completed a psychiatry internship. The MICA scores ranged from 33 to 90, with a median of 50.0 and an interquartile range of 15, indicating moderate variability. Men showed a slightly higher mean MICA score (mean rank = 47.34) than women (36.82). Students who had consulted a mental health professional had a lower mean score (37.24) than those who had never consulted (42.49). Those with a psychiatric diagnosis had a lower mean score (33.50) than those without (42.70). Students who had taken psychotropic medication showed a lower mean score (29.40) than those who had not (42.63). Similarly, participants with a family history of psychiatric disorder (mean = 34.45) or friends receiving psychiatric care (mean = 35.96) had lower scores than their counterparts (42.14 and higher). Completion of a psychiatry internship was significantly associated with lower MICA scores (p = 0.021). Furthermore, a significant difference was observed across study years, with fourth-year students having the most favorable attitudes towards mental illness (p=0.004).

**Conclusions:**

Medical students demonstrated moderately positive attitudes toward mental illness, with significant variability linked to their clinical experience. Psychiatry internships and progression in medical training were associated with more favorable attitudes, suggesting that direct contact with psychiatric patients and structured educational interventions can effectively reduce stigma and enhance empathy.

**Disclosure of Interest:**

None Declared

